# Differential miRNA Profiles Correlate With Disparate Immunity Outcomes Associated With Vaccine Immunization and Chlamydial Infection

**DOI:** 10.3389/fimmu.2021.625318

**Published:** 2021-02-22

**Authors:** Simone Howard, Shakyra Richardson, Ifeyinwa Benyeogor, Yusuf Omosun, Kamran Dye, Fnu Medhavi, Stephanie Lundy, Olayinka Adebayo, Joseph U. Igietseme, Francis O. Eko

**Affiliations:** ^1^Department of Microbiology, Biochemistry and Immunology, Morehouse School of Medicine, Atlanta, GA, United States; ^2^Winship Cancer Institute, Emory University School of Medicine, Atlanta, GA, United States; ^3^Department of Chemistry, Morehouse College, Atlanta, GA, United States; ^4^Centers for Disease Control and Prevention (CDC), Atlanta, GA, United States

**Keywords:** *Chlamydia*, immunization, infection, microRNA, immunity, pathology

## Abstract

Vaccine-induced immune responses following immunization with promising *Chlamydia* vaccines protected experimental animals from *Chlamydia-*induced upper genital tract pathologies and infertility. In contrast, primary genital infection with live *Chlamydia* does not protect against these pathologies. We hypothesized that differential miRNA profiles induced in the upper genital tracts (UGT) of mice correlate with the disparate immunity vs. pathologic outcomes associated with vaccine immunization and chlamydial infection. Thus, miRNA expression profiles in the UGT of mice after *Chlamydia* infection (Live EB) and immunization with dendritic cell (DC)-based vaccine (DC vaccine) or VCG-based vaccine (VCG vaccine) were compared using the NanoString nCounter Mouse miRNA assay. Of the 602 miRNAs differentially expressed (DE) in the UGT of immunized and infected mice, we selected 58 with counts >100 and *p*-values < 0.05 for further analysis. Interestingly, vaccine immunization and *Chlamydia* infection induced the expression of distinct miRNA profiles with a higher proportion in vaccine-immunized compared to *Chlamydia* infected mice; DC vaccine (41), VCG vaccine (23), and Live EB (15). Hierarchical clustering analysis showed notable differences in the uniquely DE miRNAs for each experimental group, with DC vaccine showing the highest number (21 up-regulated, five down-regulated), VCG vaccine (two up-regulated, five down-regulated), and live EB (two up-regulated, four down-regulated). The DC vaccine-immunized group showed the highest number (21 up-regulated and five down-regulated compared to two up-regulated and four down-regulated in the live *Chlamydia* infected group). Pathway analysis showed that the DE miRNAs target genes that regulate several biological processes and functions associated with immune response and inflammation. These results suggest that the induction of differential miRNA expression plays a significant role in the disparate immunity outcomes associated with *Chlamydia* infection and vaccination.

## Introduction

*Chlamydia trachomatis* genital infection is the commonest bacterial cause of sexually transmitted disease (STD) worldwide. Although both men and women are affected, the infection is usually more severe in women. If untreated, it can lead to irreversible complications characterized by pelvic inflammatory disease (PID), ectopic pregnancy, and tubal factor infertility (1–3). About 33% of *C. trachomatis* infection-associated PID occurs mainly in reproductive-age women and on examination, presents as pelvic or lower abdominal pain or uterine or adnexal tenderness (4, 5). Acute PID resulting from genital *Chlamydia* infection is often associated with more extended hospitalization stays, increased inflammatory markers, higher incidence of tubo-ovarian abscess, ectopic pregnancy, and tubal factor infertility (6). Tubal infertility results from tubal damage caused by inflammatory fibrosis (scarring) that lead to tubal occlusion (7). Recent studies show that *Chlamydia*-induced fibrosis and fertility-related epithelial dysfunction is associated with the pathologic process of epithelial-mesenchyme transition (EMT) (8).

There is currently no licensed human *Chlamydia* vaccine despite concerted efforts. We have developed several *Chlamydia* vaccine candidates, including a dendritic cell (DC)-based experimental cellular vaccine comprising IL-10 knockout (KO) mouse bone-marrow derived DC cultured for 12 h with UV-irradiated *Chlamydia* EBs (DC vaccine) and a *Vibrio cholerae* ghost (VCG)-based subunit vaccine consisting of the chlamydial polymorphic membrane protein D (PmpD) and porin B (PorB) proteins packaged in genetically derived *V. cholerae* cell envelops (VCG vaccine). We previously showed that immunization with DC or VCG vaccine protected mice from *Chlamydia-*induced upper genital tract (UGT) pathologies and infertility. In contrast, immunization with live *Chlamydia* elementary bodies (EBs) failed to protect against these pathologies following a subsequent genital challenge infection (9, 10). This observation is quite intriguing since immunization with these experimental vaccines and primary genital infection with live *Chlamydia* both induce a significant degree of protective immunity in the lower genital tract marked by lower microbial burden and shortened infection (9–11). Nevertheless, these studies revealed that functionally, vaccine-induced immune responses differ from *Chlamydia* infection-induced immune responses since they lead to different outcomes in the UGT. The efficacy of the DC vaccine is a function of the propensity of their antigen-presenting cells (APCs) to rapidly and preferentially activate a high magnitude of IFN-γ-secreting T cells and is associated with the induction of a persistent high Th1 cell frequency in the genital tract (11). Distinct features of *Chlamydia*-induced UGT pathology in mice are hydrosalpinx development and tubal dilatation, leading to tubal occlusion and infertility (10). We and others have shown that many of these pathological lesions occur at later time points in the absence of cultivable bacteria (10, 12). Although *C. trachomatis* infection stimulates a CD4+ T cell response to protect against reinfection (13), T cell immunity appears to be short-lived in humans. It may also influence the development of immunopathology (14).

Several mechanisms have been proposed for the inflammatory response and associated consequences in *Chlamydia* infected host cells, including the presence of chlamydial proteins and host-derived small non-coding RNAs (miRNAs) expressed during *C. trachomatis* infection (8, 15–18). MicroRNAs have emerged as important regulators of biological pathways, including immune response and inflammation by modifying gene regulatory networks (19). miRNAs are 18–22 nucleotides long, endogenous, non-coding RNAs whose primary function is RNA silencing and post-transcriptional regulation of gene expression, which is accomplished by either repressing translation or inducing mRNA degradation, depending on its sequence homology with the targeted mRNA (19, 20). Accordingly, complete complementarity between the miRNA and its target mRNA leads to cleavage and degradation, while mismatches lead to suppression of translation. Predictions by computational analysis suggest that each miRNA molecule can target 100 or more transcripts and that multiple miRNA species may regulate a single mRNA (21).

Moreover, functional studies indicate that miRNAs regulate over 30% of mammalian gene expression, involving essentially every cellular process so far investigated. These processes include cellular differentiation, maintenance of cellular integrity, development, functions and normal metabolism, reproduction, and several pathologic processes and diseases, such as fibrosis and oncogenesis (22). MiRNAs also regulate mitochondrial function, which is necessary for *C. trachomatis* development (23). The differential expression of miRNAs during ocular and genital chlamydial infection has also been severally reported (15, 16, 23–26). Specific miRNA expression can predict the development of *Chlamydia* infection-induced PID (27). The relative degree of virulence of the infecting *Chlamydia* is associated with the host miRNA expression profile, determining disease severity (18). Besides, immune cell type-specific miRNAs appear to regulate the immune response to chlamydial infection (16, 26). Thus, miRNAs play a significant role in both the quality of the host immune response and the development of complications following *Chlamydia* infection. In this study, we examined if infection with live *Chlamydia* or immunization with a VCG or DC chlamydial vaccine drives differing miRNA levels and diversity in the upper genital tract of mice. Thus, we performed a comparative assessment of the host miRNAs differentially expressed in the upper genital tract (UGT) tissues of mice after infection with live *Chlamydia* and immunization with *Chlamydia* vaccines using the NanoString nCounter Mouse miRNA assay. We showed that vaccine immunization and live *Chlamydia* infection induced a diversity of uniquely differentially expressed miRNAs in the UGT tissues of mice. In addition, we identified candidate miRNAs whose up or downregulation may account for the observed differences between live *Chlamydia* infection- and chlamydial vaccine-induced immunity.

## Materials and Methods

### Ethics Statement

In this study, the recommendations contained in the Guide for the Care and Use of Laboratory Animals of the National Institutes of Health were followed. The Institutional Animal Care and Use Committee (IACUC) of Morehouse School of Medicine (MSM) (Assurance number A3381-01) approved the study protocol (Protocol Number: 16-15). MSM-IACUC adheres to the National Institute of Health (NIH) guidelines for the care and use of laboratory animals, the Public Health Service (PHS) policy, and the Animal Welfare Act.

### Animals

Six to 7-week-old female C57BL/6J mice (stock number 000664) obtained from The Jackson Laboratory (Bar Harbor, ME) were used in this study and were allowed to acclimate for 10 days in the MSM animal facility prior to experimentation. The animals were fed food and provided water *ad-libitum* and maintained in laminar flow racks under pathogen-free conditions with a 12 h light and 12 h dark cycle. All infections, immunizations and surgery were performed under isoflurane anesthesia, and all efforts were made to minimize suffering. Mice, placed in a chamber were exposed to 2–4% isoflurane in a fume hood for 30 min. Mice were euthanized by carbon dioxide asphyxiation, followed by cervical dislocation as recommended by the Panel on Euthanasia of the American Veterinary Medical Association.

### *Chlamydia* Stocks and Antigens

In stock preparations of *Chlamydia muridarum* Nigg (the agent of mouse pneumonitis) used in this study were propagated on HeLa cell monolayers followed by purification of elementary bodies (EBs) over renografin gradients and stored at −70°C (Division of Scientific Research, Centers for Disease Control and Prevention, Atlanta), according to standard procedures. *Chlamydia* stock titers were expressed as inclusion-forming units (IFU) per milliliter (9). UV-inactivated *Chlamydia* was prepared by exposing aliquots of purified EBs in Eppendorf tubes to UV radiation for 1–2 h and stored at −70°C until used.

### Production of rVCG and IL-10KO DC Vaccines

*V. cholerae* O1 strain H1 harboring the lysis plasmid pDKLO1 was transformed with plasmids expressing pPmpD or pMAL-PorB and cultured in brain heart infusion (BHI) broth at 37°C. The rVCG vaccines were obtained by genetic inactivation of the *V. cholerae* cells as previously described (28, 29). The process involves the generation of a lysis tunnel structure through the bacterial cell envelope complex by lysis protein E, which leads to the cellular release of cytoplasm, producing cell envelopes devoid of cellular content. The culture was centrifuged to harvest the cell envelopes with the packaged PmpD and PorB proteins (VCG vaccine), which were washed, freeze-dried and stored refrigerated until used. IL-10KO DC (2 × 10^5^) were isolated from IL-10KO mice by standard protocols and pulsed for 12 h with UV-inactivated *C. muridarum* EBs (1 × 10^5^ IFU) as described previously (11). This constituted the IL-10KO DC-based cellular vaccine (DC vaccine).

### Vaccine Immunization and Chlamydia Infection of Mice

Naive female C57BL/6 wild-type mice (4/group) were immunized intranasally (IN) with 500 μg each of lyophilized rVCG-PorB and rVCG-PmpD (1 mg total) in 25 μl PBS twice 2 weeks apart. Another group (4/group) was adoptively immunized intravenously *via* the tail vein with UV-irradiated *Chlamydia* EB pulsed for 12 h with IL-10KO DC (2.5 × 10^7^ cells/mouse) (11) in 200 ml of PBS (DC vaccine). A third group of mice was infected by intravaginal administration of 1 x 10^5^ IFU of live *C. muridarum* EBs, 5 days after subcutaneous injection with 2.5 mg Depo-Provera (medroxyprogesterone acetate; Pharmacia & Upjohn Co., NY). A group immunized intravaginally with 50 μl of PBS alone served as control. Each group contained three mice and all immunizations were administered while under isoflurane (2–4%) anesthesia. Mice were euthanized 2 weeks after immunization or infection and the entire genital tract was harvested, and the cervix (lower genital tract) and uterine horns (upper genital tract) were separated and frozen immediately on dry ice and stored at −80°C.

### RNA Isolation

Harvested genital tract tissues were each homogenized using the gentleMACS Dissociator in combination with the gentleMACS C-Tubes (Miltenyi Biotech, Auburn, CA). The cellularity included in the UGT samples is distributed in the epithelial layer, lamina propria, and stroma. The predominant immune cell composition of this compartment includes T cells, macrophages/dendritic cells, natural killer (NK) cells, neutrophils, and mast cells. *Chlamydia* infects primarily epithelial cells of the genital tract and we previously showed that the inflammation/fibrosis associated with *Chlamydia*-induced UGT pathologies is preceded by pathogenic EMT, which converts normal epithelial cells into fibroblastic mesenchymal cells (8, 30). Homogenized tissue samples were treated with QIAzol Lysis reagent (Qiagen, Germantown, MD) and frozen at −80°C until RNA extraction. Total RNA was isolated using the Qiagen miRNeasy Mini Kit (Qiagen, Germantown, MD) according to the manufacturer's instructions. Residual genomic DNA was removed by DNase I treatment (ThermoFisher Scientific). RNA integrity was assessed using the Agilent Technologies- 2100 Bioanalyzer and the concentration of purified RNA was determined using the NanoDrop ND-2000 Spectrophotometer (Thermo Fisher Scientific Inc., Wilmington, DE) according to the manufacturer's instructions. RNA samples were determined to have both an A260/280 and A260/230 ratio > 1.8. All samples were analyzed in triplicates.

### MiRNA Expression Profiling

Purified RNA samples were analyzed using the NanoString nCounter Mouse miRNA Assay (NanoString Technologies, Seattle, WA). The assay involves a multiplexed hybridization reaction composed of up to 800 Reporter Probe sets (genes) and the RNA sample. Briefly, 100 ng of each RNA sample was added to the miRNA-tag ligation reaction. Following ligation, samples were diluted 1:10, and 5ul each miRNA-tag sample (equivalent to 10 ng total RNA) were added to hybridizations and subjected to 3 h of automated processing per cartridge. Data acquisition was performed on a GEN2 Digital Analyzer and processed using NanoString's data analysis application nSolver. The nCounter miRNA v3 Assays included a set of six Positive and Negative control probes to monitor hybridization efficiency, Prep Station purification, and imaging. The Positive Control Performance data was expressed as the Linearity of Counts *vs*. RNA Concentration (Supplementary Figure 1). A set of Ligation miRNA controls were added to monitor miRNA-tag ligation efficiency. Endogenous miRNA counts were used to evaluate sample quality and data normalization.

### MicroRNA Data Analysis—Normalization, Principal Components Analysis, Hierarchical Clustering

Each hybridization assay was normalized using the NanoString nSolver Analysis application based on the top-100 expressed miRNAs in each sample, filtered for those with raw counts > 20. Normalization factors ranged between 0.1 and 10 for all assays. The positive top-100 miRNA normalized counts from each group were retained for further analysis. Target identification of genes putatively regulated by miRNAs was performed using TargetScan (Diana Tools-mirPath v.3) (31). Heatmaps were generated for differentially expressed miRNAs in each immunization group using R. Overlapping expression among miRNAs identified following immunization with the different vaccines was visualized using Venny 2.0, an interactive Venn diagram tool for comparing name lists of up to four genomic datasets (32). Subsequent Pathway, Core and Comparison analyses for all the false discovery rate (FDR)-corrected differentially expressed miRNAs in infected and immunized mice were analyzed using the Ingenuity Pathway Analysis (IPA) software (Qiagen Inc.) and Diana MirPath (33). The top networks, molecular and cellular functions, diseases and disorders and physiological system development and functions were determined using Ingenuity System Interactive Pathway Systems (version 18,488,943). Stringency was set at “highly predicted” and “experimentally validated.” The software uses its own internal algorithm and other databases, including TarBase, TargetScan, and microT-CDS, as well as findings published in the literature. Complete reference set for this analysis was carried out using the Ingenuity Knowledge Base (Genes + Endogenous Chemicals) for all molecules associated with diseases, functions, pathways, or list annotations.

### qRT-PCR

Total RNA was isolated as indicated above and cDNA was synthesized using the miScript II RT Kit (Qiagen, Germantown, MD). qRT- PCR was performed on BioRad CFX96 system (Bio-Rad, Hercules, CA) using specific miRNA primers and miScript SYBR Green PCR Kit (Qiagen) according to the manufacturer's protocol. The relative fold changes in miRNA expression were calculated by the delta-delta-cycle threshold (ΔΔCt) method comparing miRNA expression levels in the genital tracts after immunization with the different vaccines. U6 miRNA was used as an endogenous control for expression normalization, and ΔΔCt was calculated as the difference between immunized and non-immunized ΔCt. The results are expressed as fold change, (the mean ± *SD* of three independent qRT-PCR runs), corresponding to 2^−ΔΔCt^.

### Statistical Analysis

The statistical analysis was performed using GraphPad Prism 8 software for Mac (GraphPad Software, Inc., La Jolla, CA, USA). Quantitative data were expressed as the mean ± standard deviation (*SD*), and the error bars indicate the SD from the mean. The statistical significance of the quantitative differences between any two groups was determined by the Student's two-tailed *t*-test, and between three groups was assessed by analysis of variance (ANOVA). Statistical significance was determined at *p*-values < 0.05.

## Results

### Profile of MiRNAs Expressed in the Upper Genital Tracts of Mice After Immunization and Infection

**Figure 1:**
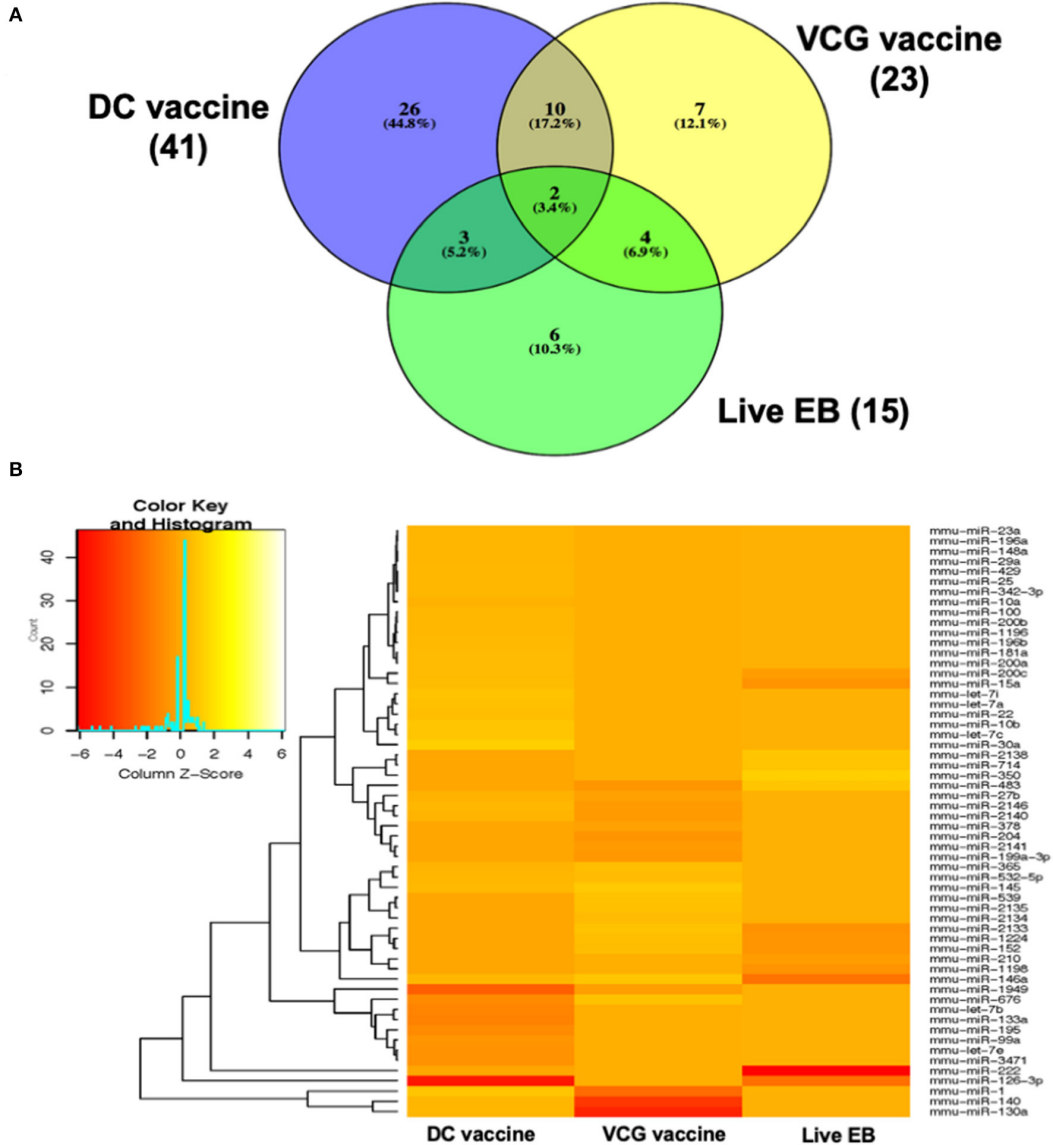
Cluster and Heat map analysis of miRNAs significantly differentially expressed in UGT of mice after vaccine immunization and live *Chlamydia* infection. **(A)** Venn diagram of the top 58 upregulated and downregulated differentially expressed miRNAs after DC vaccine (41) or VCG vaccine (23) immunization and live *Chlamydia* infection (15). The numbers indicate the unique and common miRNAs in the immunization and infection groups. **(B)** Hierarchical cluster analysis of the top 58 upregulated and downregulated differentially expressed miRNAs after vaccine immunization and live *Chlamydia* infection. Each row represents a miRNA and each column, an immunization or infection group. On the scale of fold regulation, yellow corresponds to upregulated miRNAs, red corresponds to downregulated miRNAs, and brown indicates no expression difference.

MicroRNAs (mRNAs) with altered expression in the upper genital tracts of vaccine-immunized and live *Chlamydia*-infected mice were determined using a Nanostring quantitative assay platform. A total of 602 miRNAs were differentially expressed (DE) in the genital tracts of immunized and infected mice as compared to non-infected tissues. Of these, 58 miRNAs with counts >100 and *p*-values < 0.05 were selected for further analysis. The proportion of miRNAs detected showed 41 (71%) were detected in DC vaccine immunized mice, 23 (40%) in VCG vaccine immunized mice, and 15 (26%) in *Chlamydia* infected mice. Comparison of the 58 selected miRNAs using a Venn Diagram showed that only two miRNAs were common among the three groups, three between the DC vaccine and live *Chlamydia* infection groups and four between the VCG vaccine and *Chlamydia* infection groups (Figure 1A). Interestingly, we found that 10 DE miRNAs were common between the DC and VCG vaccine groups (Figure 1A). The hierarchical clustering of the 58 differentially regulated miRNAs is shown in Figure 1B. Clustering was based on significance levels, with lighter colors representing higher significance values. In the DC vaccine group, miR-196b-5p and miR-3471 clustered together while miR-181a-5p and miR-10b-5p clustered together. In the VCG vaccine group, miR-539-5p and miR-2135 clustered together and were the most significantly upregulated miRNAs, while miR-350-3p and miR-222-3p clustered together in the live EB infected group. The clustering together of these miRNAs suggests they may exhibit similar pathway targeting patterns. The results further indicate that the diversity of up- or down-regulated miRNA was different for each immunization and infection group (Tables 1–3). Thus, for the DC vaccine group, 32 miRNAs were upregulated, while nine were downregulated. Twelve miRNAs were upregulated and 11 were downregulated in the VCG vaccine group. In contrast with the DC and VCG vaccine groups, only four miRNAs were up-regulated and 11 were down-regulated in the live EB infection group. These results indicate that vaccine immunization and *Chlamydia* infection induces the DE of distinct miRNA profiles in the UGT of mice.

**Table 1:** miRNAs DE expressed between DC-based vaccine and control.

**miRNA**	**Expression**	**Fold change**	*P*-value^*^
**Upregulated (32)**			
mmu-miR-1a-3p		2.17	0.0196
^*^mmu-let-7a-5p		1.22	0.0344
^*^mmu-let-7b-5p		1.38	0.0085
^*^mmu-let-7c-5p		1.48	0.0039
^*^mmu-let-7e-5p		1.45	0.0190
^*^mmu-let-7i-5p		1.62	0.0155
^*^mmu-miR-10a-5p		1.45	0.0279
^*^mmu-miR-10b-5p		2.35	0.0003
^*^mmu-miR-22-3p		1.70	0.0129
^*^mmu-miR-23a-3p		1.18	0.0024
^*^mmu-miR-25-3p		1.37	0.0229
mmu-miR-27b-3p		2.76	0.0013
^*^mmu-miR-29a-3p		1.33	0.0057
^*^mmu-miR-30a-5p		1.26	0.0168
^*^mmu-miR-99a-5p		1.65	0.0017
^*^mmu-miR-100-5p		1.58	0.0031
mmu-miR-126-3p		1.41	0.0129
mmu-miR-130a-3p		1.44	0.0394
^*^mmu-miR-133a-3p		1.47	0.0207
mmu-miR-140-5p		1.41	0.0203
mmu-miR-145-5p		1.85	0.0164
mmu-miR-146a-5p		1.87	0.0116
^*^mmu-miR-181a-5p		2.52	0.0012
^*^mmu-miR-195a-5p		1.11	0.0224
^*^mmu-miR-196a-5p		2.04	0.0093
^*^mmu-miR-196b-5p		2.84	0.0029
^*^mmu-miR-342-3p		1.47	0.0379
mmu-miR-532-5p		1.47	0.0080
mmu-miR-365-3p		1.83	0.0155
mmu-miR-676-3p		1.74	0.0057
mmu-miR-1949		2.72	0.0009
^*^mmu-miR-3471		3.67	0.0256
**Downregulated (9)**			
mmu-miR-15a-5p		−1.43	0.0500
^*^mmu-miR-148a-3p		−1.63	0.0010
^*^mmu-miR-200a-3p		−3.11	0.0056
^*^mmu-miR-200b-3p		−2.22	0.0123
mmu-miR-200c-3p	Anti-inflammatory	−2.37	0.0467
^*^mmu-miR-429-3p		−2.85	0.0007
^*^mmu-miR-1196		−1.66	0.0322
mmu-miR-2140		−6.02	0.0118
mmu-miR-2146		−12.21	0.0034

* *Expressed in DC vaccine only*.

**Table 2:** miRNAs DE expressed between VCG-based vaccine and control.

**miRNA**	**Expression**	**Fold change**	*P*-value^*^
**Upregulated (12)**			
mmu-miR-1a-3p		2.17	0.0196
mmu-miR-1a-3p		1.73	0.0486
mmu-miR-27b-3p		2.30	0.0032
mmu-miR-130a-3p		1.22	0.0113
mmu-miR-140-5p		1.28	0.0476
mmu-miR-145a-5p		1.53	0.0414
mmu-miR-146a-5p		1.53	0.0189
mmu-miR-532-5p		1.31	0.0051
^*^mmu-miR-539-5p		1.88	0.0105
mmu-miR-365-3p		1.81	0.0484
mmu-miR-676-3p		1.56	0.0014
mmu-miR-1949		2.54	0.0031
^*^mmu-miR-2135		1.66	0.0128
**Downregulated (11)**			
mmu-miR-152-3p		−1.47	0.0053
^*^mmu-miR-199a-3p		−1.31	0.0419
^*^mmu-miR-204-5p		−2.24	0.0060
^*^mmu-miR-378a-3p		−1.24	0.0242
mmu-miR-483-5p		−2.04	0.0187
mmu-miR-1224-5p		−2.49	0.0427
mmu-miR-2133		−13.56	0.0018
^*^mmu-miR-2134		−2.53	0.0067
mmu-miR-2140		−6.79	0.0002
^*^mmu-miR-2141		−2.22	0.0241
mmu-miR-2146		−11.69	0.0021

* *Expressed in VCG vaccine only*.

**Table 3:** miRNAs DE expressed between *Chlamydia* infection and control.

**miRNA**	**Expression**	**Fold change**	*P*-value^*^
**Upregulated (4)**			
mmu-miR-126a-3p		1.31	0.0456
mmu-miR-146a-5p		1.42	0.0347
^*^mmu-miR-222-3p		1.52	0.0452
^*^mmu-miR-350-3p		2.17	0.0437
**Downregulated (11)**			
mmu-miR-15a-5p		−1.84	0.0036
mmu-miR-152-3p		−1.31	0.0261
mmu-miR-200c-3p		−2.00	0.0239
^*^mmu-miR-210-3p		−1.98	0.0033
mmu-miR-483-5p		−1.86	0.0191
^*^mmu-miR-714		−2.02	0.0305
^*^mmu-miR-1198-5p		−1.34	0.0287
mmu-miR-1224-5p		−4.03	0.0118
mmu-miR-2133		−12.16	0.0404
^*^mmu-miR-2138		−2.96	0.0366
mmu-miR-2140		−4.43	0.0075

* *Expressed in live Chlamydia infection only*.

### Pattern of Up- and Down-Regulated MiRNAs Uniquely DE in the Upper Genital Tracts of Immunized and Infected Mice

**Figure 2:**
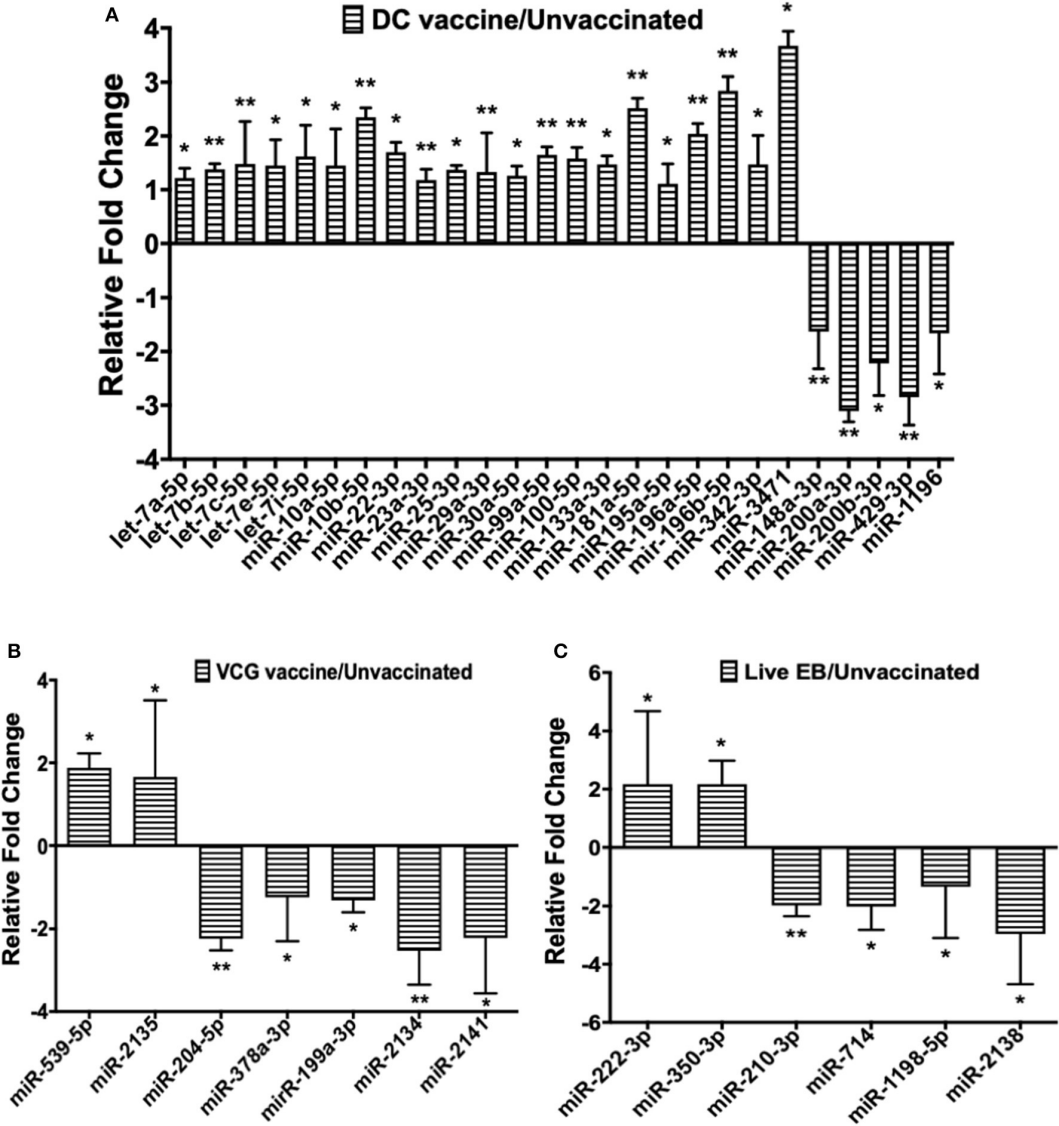
Quantification of miRNAs uniquely differentially expressed in the UGT of mice after **(A)** DC vaccine immunization, **(B)** VCG vaccine immunization, and **(C)** live *Chlamydia* infection. miRNAs were normalized using the NanoString nSolver Analysis application based on the top-100 expressed miRNAs in each sample, filtered for those with raw counts > 20. Each reaction was performed with triplicate samples, and the data is shown as the mean for each group. Results are expressed as relative fold changes in miRNA expression compared to non-immunized controls. Statistical analysis was performed by the NanoString nSolver Analysis software and significant levels were simultaneously generated with the data. *p < 0.05, **p < 0.01.

Twenty-one of the 32 up-regulated (including five members of the Let-7 family) and five of the nine down-regulated miRNAs were unique to the DC vaccinated mice (Figure 2A). On the other hand, only two of the 12 up-regulated and five of the 11 down-regulated miRNAs were unique to the VCG vaccinated mice (Figure 2B). Among these, miR-2135 (up-regulated) and miR-2134 (down-regulated) have not previously been reported in the literature. In addition, two of the four up-regulated and four of the 11 down-regulated miRNAs were unique to the live EB group (Figure 2C). Many of these miRNAs with log fold changes ranging from−13.56 to 3.67 have been shown to have human homologs. Comparative profiling also showed that nine of the upregulated (let-7a-5p, let-7c-5p, let-7e-5p, miR-23a-3p, miR-25a-3p, miR-29a-3p, miR-181a-5p, miR-195a-5p, and miR-196a-5p) and 4 of the downregulated (miR-148a-3p, miR-200a-3p, miR-200b-3p, and miR-429-3p) miRs uniquely DE in the UGT of DC vaccine-immunized mice were inflammation and immunopathology-associated (Table 4) (8, 30, 35). The results revealed that many of the uniquely DE miRNAs in the UGT of DC vaccine-immunized mice were associated with inflammation and immunopathology.

**Table 4:** Inflammation and immunity-associated miRNAs uniquely differentially expressed (DE) in the UGT of DC vaccine-immunized mice.

**DE miRNA**	**Fold-change**	**Targets**	***P*-value**	**Biological function/disease**	**Anti/pro-inflammatory**	**References**
let-7a-5p	1.22	IL-6, HMGA1/2	0.0344	Downregulating Inflammation	Anti-Inflammatory	(34)
Let-7c-5p	1.48	Caspase 3, N-RAS, MMP1	0.0039	Downregulating Inflammation	Anti-Inflammatory	(35, 36)
Let-7e-5p	1.45	TLR4	0.0190	Downregulating Inflammation	Anti-Inflammatory	(37)
miR-23a-3p	1.18	1KKα CiGadd45ab	0.0024	Innate immune responses Inflammation/Apoptosis	Anti-Inflammatory Pro-Inflammatory	(37, 38)
miR-25-3p	1.37	Adam10	0.0229	Downregulating inflammation	Anti-Inflammatory	(39)
miR-29a-3p	1.33	TET1, MUC1 TRIM68, CDC4 MMP2	0.0057	Inflammation/tumor suppressor Cell migration/proliferation Apoptosis	Anti-Inflammatory - -	(40)
miR-181a-5p	2.52	MMP-14 Ago2, Smad2/3	0.0012	Innate immune responses Cell migration/proliferation EMT/tumor suppressor	Anti-Inflammatory Anti-Inflammatory Pro-inflammatory	(30, 41, 42)
miR-195a-5p	1.11	Smad7, TRIM14 Cyclin E1	0.0224	Cell proliferation/migration Tumor suppressor	- -	(43)
miR-196a-5p	2.04	MAP4K3, IKKα ELOVL1	0.0093	Downregulating inflammation Migration/EMT	Anti-Inflammatory -	(44)
miR-148a-3p	−1.63	PTEN/AKT	0.0010	Downregulating inflammation	Anti-Inflammatory	(45)
miR-200a-3p	−3.11	E-cadherin, QKI, ZEB1/2	0.0056	EMT induction Cell proliferation/migration	Pro-Inflammatory -	(46, 47)
miR-200b-3p	−2.22	E-cadherin, QKI, ZEB1/2	0.0123	EMT induction	Pro-Inflammatory	(8, 48)
miR-429-3p	−2.85	ZEB1 CRKL	0.0007	EMT induction Cell proliferation/migration	Pro-Inflammatory -	(8, 49)

### Pattern of Up- and Down-Regulated DE MiRNAs Common Between Infected and the Different Immunization Groups

**Figure 3:**
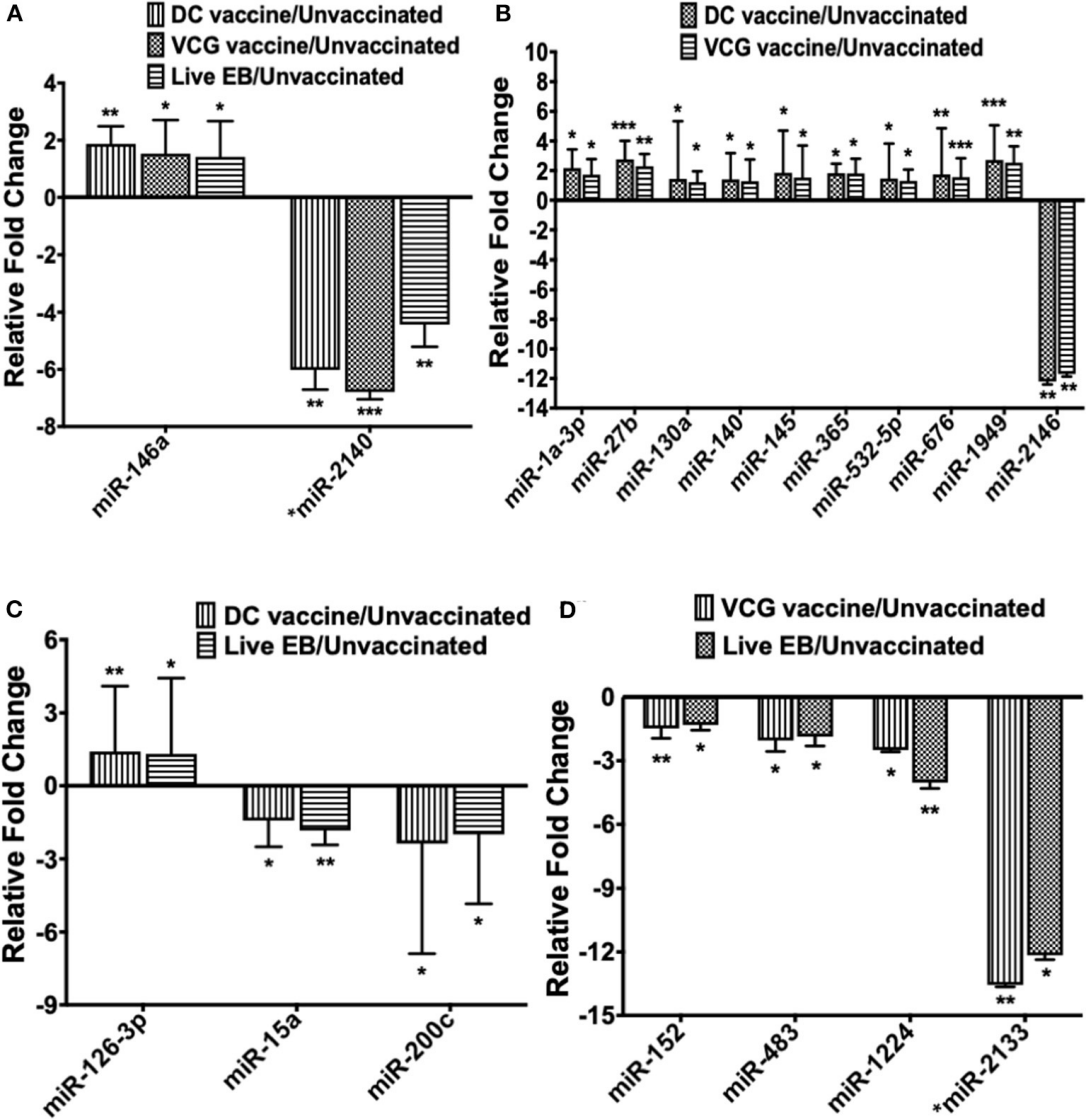
The pattern of DE miRNAs in UGT of mice common between infected and immunized mice. **(A)** Levels of differentially expressed miRNA common between the immunization (DC and VCG vaccines) and live *Chlamydia* infection groups. **(B)** Levels of differentially expressed miRNA common between DC and VCG vaccine groups. **(C)** Levels of differentially expressed miRNA common between DC vaccine-immunized and live *Chlamydia* infection groups. **(D)** Levels of differentially expressed miRNA common between VCG vaccine-immunized and live *Chlamydia* infection groups. Data, which are the means from three animals per group, are plotted as fold change relative to non-immunized control levels. Significant differences between three groups were evaluated by one-way analysis of variance (ANOVA) with Tukey's post multiple comparison test and between two groups by Student's *t*-test at *p < 0.05, **p < 0.01, ***p < 0.001.

Only one inflammation and immunopathology-associated miRNA (miR-146a) was commonly expressed among the immunized and infected groups (Figure 3A). In addition, four inflammation and immunopathology-associated miRs (miR-27b, miR-130a, miR-140, and miR-145) were commonly expressed in the genital tracts of DC and VCG vaccine immunized mice (Figure 3B). Of the two miRNAs commonly expressed in the genital tracts of infected and immunization groups, miR-146a was up-regulated while miR-2140, which has only previously been reported in the salivary glands of ticks after a blood meal (50), was down-regulated (Figure 3A). Among the 10 miRNAs (mmu-miR-1a-3p, mmu-miR-130a-3p, mmu-miR-140-5p, mmu-miR-145-5p, mmu-miR-1949, mmu-miR-2146, mmu-miR-27b-3p mmu-miR-365-3p, mmu-miR-532-5p, and mmu-miR-676-3p) commonly expressed in DC and VCG vaccine immunized mice, only mmu-miR-2146 was down-regulated while the rest were up-regulated (Figure 3B). Of the three miRNAs commonly expressed in the genital tracts of DC vaccine immunized and *Chlamydia* infected mice, miR-126-3p was up-regulated while miR-15a and miR-200c were down-regulated (Figure 3C). All four miRNAs (miR-1224, miR-152, miR-2133, and miR-483) commonly expressed in the VCG vaccine-immunized and *Chlamydia* infected mice were down-regulated (Figure 3D). Our findings reveal that five of the DE miRNAs (miR-2134, miR-2135, miR-2138, miR-2140, and miR-2146) are novel; these have not previously been characterized in the context of infection or vaccination.

### qPCR Data Validation

**Figure 4:**
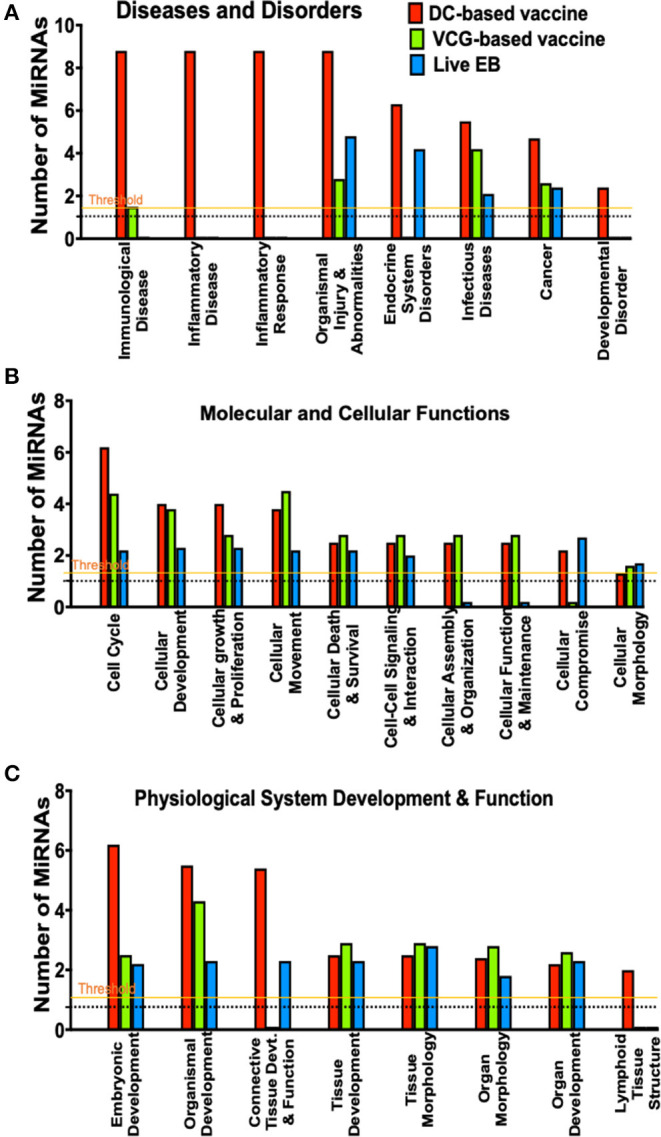
A comparison of canonical pathways predicted to be regulated by miRNAs differentially expressed in the UGT of mice after immunization and live *Chlamydia* infection. **(A)** Pathways predicted to regulate diseases and disorders after vaccine immunization and *Chlamydia* infection. **(B)** Pathways predicted to regulate molecular and cellular functions after vaccine immunization and *Chlamydia* infection. **(C)** Pathways predicted to regulate physiological system development and function after vaccine immunization and *Chlamydia* infection.

To validate the differential expression of miRNAs identified by the Nanostring quantitative assay platform, we selected four DE miRNAs (miR-146, 126, 15a, and 200c) for qPCR analysis. The miR-146 was selected from the two miRNAs that were commonly expressed in all groups of mice after immunization and infection. The remaining three miRNAs were those that were common between the DC vaccine and *Chlamydia* infected groups. The results showed that the data generated by the Nanostring assay and qPCR were consistent in terms of up- or down-regulation and differed only in the magnitude of the response measured by the two approaches (Figure 4 and Supplementary Figure 2).

### MiRNAs DE in the Upper Genital Tracts of Mice After Infection and Immunization Regulate a Number of Pathways Involved in Disease and Biological Functions

To evaluate the biological pathways activated in the upper genital tracts of mice following infection and immunization, we performed core analysis of the DE miRNAs in the framework of biological processes and molecular networks (diseases and disorders, molecular and cellular functions, and physiological system development) using IPA. In pathways associated with diseases and disorders, there were substantial differences in the number of differentially expressed miRNAs regulating immunological and inflammatory diseases, inflammatory response, endocrine system disorders, infectious diseases, cancer and developmental disorder after *Chlamydia* infection and immunization with DC and VCG vaccines (Figure 4A). In the case of molecular and cellular functions-associated pathways, there were differences in the number of differentially expressed miRNAs regulating cell cycle, cellular development, cellular growth and proliferation, cellular movement, cellular assembly and organization, cellular function and maintenance, and cellular compromise between the infection and immunization groups (Figure 4B). Also, there were substantial differences in the number of differentially expressed miRNAs regulating embryonic development, organismal development, connective tissue development and function, and lymphoid tissue structure pathways associated with physiological system development and function after *Chlamydia* infection and immunization with DC and VCG vaccines (Figure 4C).

**Figure 5:**
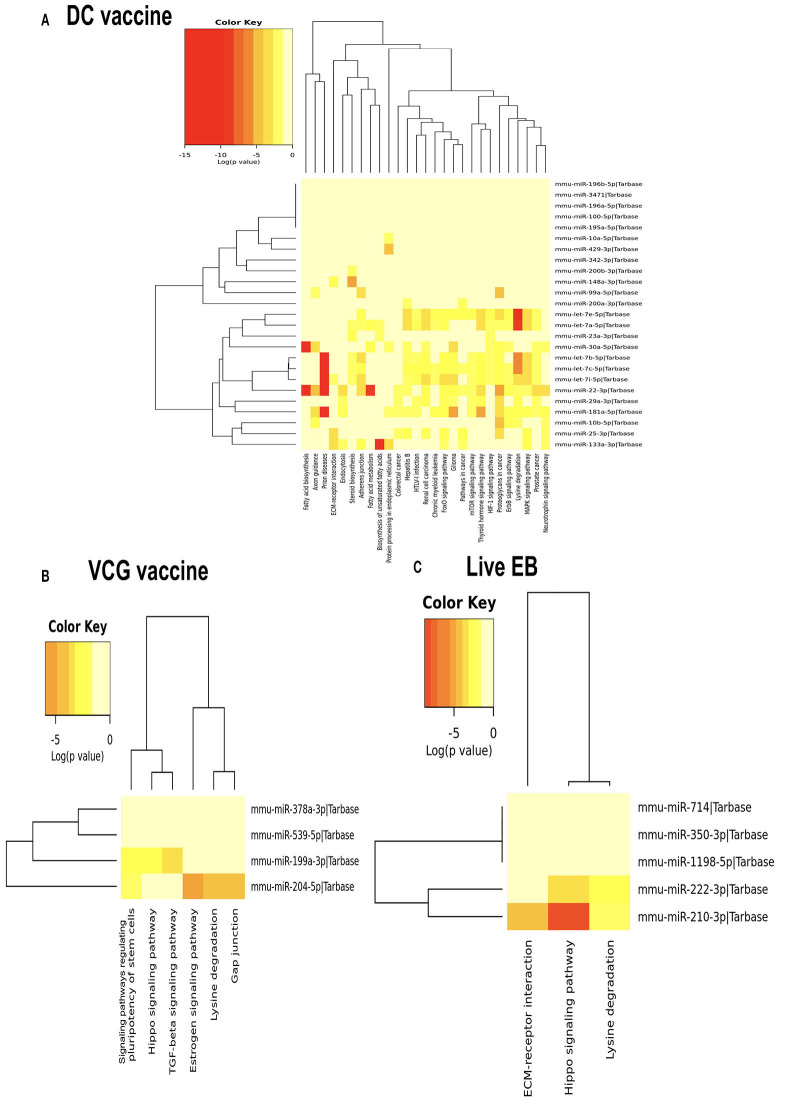
Significance cluster analysis showing pathways predicted to be regulated by miRNAs differentially expressed in the UGT of mice after immunization with DC vaccine **(A)**, VCG vaccine **(B)**, and live *Chlamydia* infection **(C)**. The hierarchical clustering results for miRNAs are shown on the right Y-axis and pathways on the X-axis. On the miRNA axis, we can identify miRNAs clustered together by exhibiting similar pathway targeting patterns. Similar clustering can also be seen on the pathway axis. Darker colors represent lower significance values.

We generated KEGG pathways of the differentially expressed miRNAs unique to each infection and immunization group using the Diana Tools-mirPath v.3 (33). Based on the DIANA-TarBase reference database, a higher number of biological pathways were predicted to be targeted by miRNAs differentially expressed in the UGT of mice after immunization with the DC vaccine compared to the VCG vaccine and *Chlamydia* infection groups. For the DC vaccine group, 27 pathways were predicted to be regulated by the DE miRNAs (Figure 5A). The pathways included MAPK, ErbB, HIF-1B, mTOR, Pathways in cancer, Glioma, FoxO, Chronic myeloid leukemia, Renal cell carcinoma, Hepatitis B, Colorectal cancer, Protein processing in the endoplasmic reticulum, Fatty acid metabolism, ECM-receptor interaction, and Fatty acid biosynthesis. For the VCG vaccine group, six pathways were predicted to be regulated by the differentially expressed miRNAs (Figure 5B), including signaling pathways regulating pluripotency of stem cells, Hippo signaling, TGF-beta signaling, Estrogen signaling, lysine degradation, and gap junction. For the *Chlamydia* infection group, three pathways were predicted to be regulated by the differentially expressed miRNAs (Figure 5C) and included lysine degradation, Hippo signaling and ECM-receptor interaction. Of these biological pathways, the mTOR, FoxO, MAPK, HIF-1, ErbB, Hippo, TGF-beta, ECM-receptor interaction, and Estrogen signaling pathways were identified as important markers of immunity and inflammation. We selected specific KEGG pathways regulated by miRNAs DE in the upper genital tract in response to DC vaccine immunization to buttress the significance of these pathways in immune response, host cell modification, and cell to cell communication (Supplementary Figures 3A–G).

**Figure 6:**
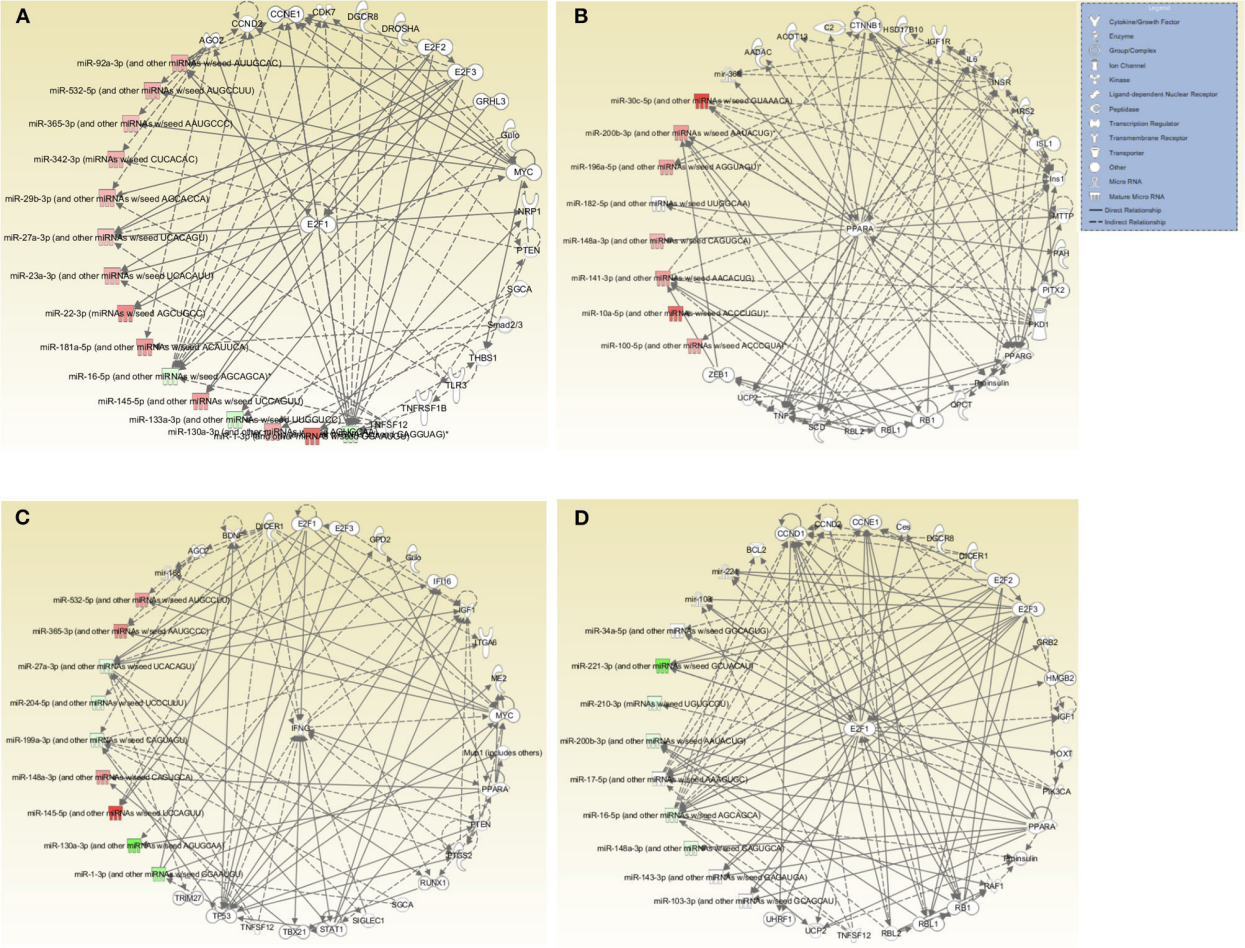
Networks associated with differentially expressed miRNAs. **(A)** Network One - Cancer, Organismal Injury and Abnormalities, and Cell Cycle associated with miRNAs DE in the genital tract of mice immunized with DC-based Vaccine. E2F1, a transcription regulator, is the central molecule associated with miRNAs. **(B)** Network Two - Energy Production, Lipid Metabolism, Small Molecule Biochemistry associated with miRNAs DE in the genital tract of mice immunized with the DC-based Vaccine. The transcription regulator, PPARA, is the central molecule associated with miRNAs. **(C)** Cellular Development, Cellular Growth and Proliferation and Organ Development Network associated with miRNAs DE in the genital tract of mice immunized with the VCG-based *Chlamydia* Vaccine. IFNG, a cytokine/growth factor, is the central molecule associated with miRNAs. **(D)** Cell Cycle, Embryonic Development, Connective Tissue Development and Function Network associated with miRNAs DE in the genital tract of mice infected with Live *Chlamydia*. The transcription regulator E2F1 is the central molecule associated with miRNAs.

Further evaluation of the top networks associated with differentially expressed miRNAs using IPA showed that for the DC vaccine, two networks were generated; Network 1 consists of DE miRNAs and 15 focus molecules associated with Cancer, Organismal Injury and Abnormalities, and Cell Cycle. E2F Transcription Factor 1 (E2F1), a transcription regulator, was the central focus molecule associated with the miRNAs (Figure 6A). Network 2 consists of miRNAs and molecules associated with Energy Production, Lipid Metabolism, and Small Molecule Biochemistry. There were seven focus molecules, with the transcription regulator, Peroxisome Proliferator-Activated Receptor Alpha (PPARA) being the central focus molecule associated with miRNAs (Figure 6B). The VCG vaccine generated one associated network consisting of DE miRNAs and molecules associated with Cellular Development, Cellular Growth and Proliferation, and Organ Development with nine focus molecules. Interferon-gamma (IFNG), a cytokine/growth factor, was the central molecule associated with miRNAs (Figure 6C). The *Chlamydia* infection group generated one associated network consisting of Cell Cycle, Connective Tissue Development and Function, and Embryonic Development. There were 34 focus molecules and the transcription regulator, E2F1, was the central molecule associated with miRNAs (Figure 6D). Together, these results demonstrate that miRNAs DE in the upper genital tracts of mice after infection and immunization regulate pathways involved in disease and biological functions.

## Discussion

We investigated if the differential miRNA profiles induced in the UGT of mice correlate with the disparate immunity vs. pathologic outcomes associated with vaccine immunization and chlamydial infection. The study shows that immunization with select chlamydial vaccines or infection with live *Chlamydia* drives differing miRNA levels and diversity in the UGT of mice. Among the miRNAs uniquely expressed in each of the experimental groups, we observed that miRNAs elicited by the DC vaccine were more likely to be upregulated than down regulated. In contrast, miRNAs in the infection group were more likely to be down regulated. The finding of significant differences in the number and diversity of up- and down-regulated miRNAs uniquely DE in the upper genital tracts of vaccine immunized and *Chlamydia* infected mice indicates that the miRNA profile expressed by immunization differs substantially from that expressed following infection. Differential expression of miRNAs during *Chlamydia* genital infection has previously been associated with immune response and pathogenesis (8, 16, 18, 24, 26).

Interestingly, the two miRNAs common to all the three treatment groups were all differentially expressed in the same direction, indicating that these miRNAs may be significant in some of the changes associated with protection against pathology. One of these miRNAs, miR-146a, is a crucial regulator of the innate immune response whose overexpression suppresses hepatic stellate cell proliferation and activation and inhibits LPS-induced pro-inflammatory cytokine secretion (51). Also, miR-146a attenuates liver fibrosis by directly suppressing the profibrogenic effects of transforming growth factor-β1 (TGFβ1) (52). Our finding of the expression of miR-2134, miR-2135, miR-2138, miR-2140, and miR-2146 following infection or vaccination is novel. The only report of miR-2140 has been in the salivary glands of ticks after a blood meal (50). In general, all the differentially expressed miRNAs common to the vaccine groups were in the same direction, either upregulated or downregulated, implying that they are likely to regulate the same pathways. It also appears differences between the vaccines are more in the nature or unique miRNAs found in each vaccine group than in similar groups. Using four select miRNAs, we also validated the miRNA expression changes identified by the Nanostring quantitative assay platform, demonstrating a strong correlation between NanoString and the qPCR methods.

We observed the differential expression of many inflammation and immunopathology-associated miRNAs following immunization and infection. The role of these miRNAs in bacterial infectious diseases involves the modulation of inflammatory responses, tissue remodeling, and innate and adaptive immunity (53). Increasing evidence shows that inflammatory-related miRNAs play essential roles in maintaining fertility formation, eliminating cancer, and development of the male reproductive tract (54). One of the inflammation-associated miRNAs, miR-146a, is induced by different pro-inflammatory stimuli, such as IL-1β and TNF-α (55, 56), and is upregulated in various human pathologies associated with activation of inflammatory responses (57, 58). Also, the expression of miR-146a reportedly increased after *Helicobacter pylori* infection in an NF-κB-dependent manner (53). This increase may inhibit the expression of IL-8, macrophage inflammatory protein (MIP)-3α, TNF-α, and IL1β by reducing NF-κB activity (59, 60). These strongly support the role of miR-146 in the regulation of inflammation and may represent a potential tool for therapeutic intervention in inflammatory mediated pathological disease conditions. Other inflammation and immunopathology-associated miRNAs uniquely DE in the UGT of DC vaccine-immunized mice, including miR-23a-3p, miR-25a-3p, and miR-29a-3p have been reported to be involved in the induction of innate immune responses, inflammation, cell migration/proliferation, apoptosis, and tumor suppression (37–40).

Of the biological pathways predicted by KEGG pathway analysis to be targeted by miRNAs differentially expressed after immunization and infection, mTOR, FoxO, MAPK, HIF-1, ErbB, Hippo, TGF-beta, ECM-receptor interaction, and Estrogen signaling pathways are significant markers of immunity and inflammation. We previously showed that downregulation of certain miRNAs, such as miR-15a and miR-27a targeted pathways, including the TGF-beta pathway to prevent fibrogenesis (30). Here, the TGF-beta pathway was predicted to be regulated by miR-204-5p, which was uniquely DE (downregulated) following VCG vaccine immunization. It is therefore conceivable that miR-204-5p controls fibrosis and maintains epithelial integrity in the UGT by specifically targeting TGF-beta signaling. Thus, although TGF-beta is known to play a crucial role in the pathogenesis of fibrosis, it also plays a significant role in tissue homeostasis, immunity and cell proliferation (61). Previous studies reported that the Forkhead transcription factor 1 (FoxO1) regulates genes involved in apoptosis and autophagy, anti-oxidative enzymes, cell cycle arrest genes, and metabolic and immune regulators (62, 63). The FoxO1/3 proteins are well-established anti-proliferation and proapoptotic factors that have favorable inhibitory effects on fibroblast activation and subsequent extracellular matrix (ECM) production capable of ameliorating fibrosis in organs such as the heart, liver, lungs, and kidneys (64). Therefore, it has been suggested that inhibition of fibroblast activation may be a promising anti-fibrosis therapeutic strategy (65). Fibrosis is the excessive formation of fibrous connective tissue (66) resulting from copious amounts of extracellular matrix (ECM) molecules and is essential for wound healing and maintenance of structural integrity (67, 68). FoxO and ECM production, known to prevent *Chlamydia*-induced fibrosis and infertility, are two biological pathways predicted in our study to be targeted by DC vaccine-induced miRNAs (8, 9). An additional interesting finding from this study is the observation that the mammalian target of rapamycin (mTOR) pathway was only targeted by DC vaccine-induced miRNAs. The beneficial role of mTOR in modulating inflammation and autophagy under liver ischemia/reperfusion (IR) injury has recently been reviewed (69). mTOR is an evolutionarily conserved serine/threonine protein kinase that plays a vital role in regulating mRNA translation, metabolism, and protein turnover (70). An excessive inflammatory response is a crucial mechanism of liver IR injury, and overexpression of mTOR in the liver significantly reduces liver inflammation and apoptosis (71). Furthermore, mTOR-deficient mice express higher levels of inflammation-related genes such as MCP-1, TNF-α, and IL-6 than wild-type mice after liver IR by negatively modulating NF-κB (71). These results suggest that the FoxO, mTOR, and ECM pathways or miRNAs targeting them might be vital targets for the therapy and prevention of *Chlamydia*-induced fibrosis, which subsequently leads to infertility and could guide the development of therapeutic agents for ameliorating *Chlamydia*-induced fibrosis in the female upper genital tract.

Several genes were directly or indirectly regulated by several miRNAs, some of which are associated with inflammation and immunopathology. For example, Ago2 and Smad2/3 were predicted targets of miR-181a-5p, and loss of Smad3 blocks EMT, leading to fibrotic sequelae (30). miR-181a, a member of the miR-181 family, regulates immune function and plays a significant role in innate and adaptive immune responses (41). In cancer studies, miR-181a-5p inhibits cancer cell migration and angiogenesis *via* downregulation of matrix metalloproteinase-14 (MMP-14) (42). More recently, miR-181a was reported to be involved in the modulation of CD4^+^ T cell activation and plasticity and decreased the expression level of the Th1-related transcription factor T-bet and the Th17-related transcription factor RORγt (72). Also, mir-204-5p, which was downregulated following immunization with VCG vaccine was associated with Dicer 1, a ribonuclease III enzyme whose loss in mouse oviducts results in significant reproductive abnormalities (15). *Chlamydia* infection alters the expression of crucial miRNAs that regulate the integrity of Dicer, EMT, fibrosis, and tumorigenesis in the reproductive system (8, 15). We recently showed that the upregulation of mir-204-5p following *Chlamydia* infection leads to genital tract pathology (23). Another miRNA, miR-146a, in addition to being directly regulated by the E2F2 and E2F3 transcription factors, was also indirectly regulated by Dicer 1. miR-146a, a member of the inflammation-inducible miRNA family, negatively regulates Toll-like receptor (TLR) signaling (73) and has been reported to modulate innate immune responses during bacterial infection by targeting IRAK1 and TRAF6 (55). It also negatively regulated NF-κB activation by inhibiting IRAK1 and TRAF6 expression, significantly inhibiting breast tumor growth (74). The dysregulation of these miRNAs following vaccine immunization strongly suggests their involvement in protection against UGT pathology by regulating Dicer 1 expression. ZEB1, RBL1, RB1, and PPARA were direct targets of miR-200b-3p. Previous studies have described the role of miR-200 family members, including miR-200a, miR-200b, miR-200c, and miR-429-3p in the regulation of expression of the E-cadherin transcriptional repressors ZEB1, ZEB2, and CRKL previously implicated in EMT and tumor metastasis (8, 46–49). We previously showed that the downregulation of miR-200a, miR-200b, miR-200c, and miR-429-3p following genital *Chlamydia* infection resulted in EMT induction by targeting ZEB1 (8). Our study confirms the downregulation of miR-200c after *Chlamydia* infection and further shows that immunization with the DC vaccine also downregulates miR-200a, miR-200b, miR-200c, and miR-429-3p. Thus, the miR-200 family members act as negative regulators of ZEB1, RBL1, RB1, and PPARA expression leading to processes that contribute to the disruption of the epithelial functional integrity of the UGT and induction of EMT and fibrosis. Thus, EMT, in addition to tumor invasion and metastasis, also plays a crucial role in embryonic development, wound healing, and tissue fibrosis. We found that Ago2 and CCNE1 were direct targets of members of the Let-7 family that were upregulated following immunization with the DC vaccine. Several members of the Let-7 family, including let-7a-5p, let-7c-5p, and let-7e-5p have previously been reported to inhibit migration, invasion and EMT by targeting IL-6, HMGA1/2, Caspase 3, N-RAS, MMP1, and TLR4 (34–37). Although our study did not identify any direct targets for miR-195a-5p and IGF1R and INSR were only indirectly associated with miR-196a-5p, previous studies have reported the involvement of these miRs with cell proliferation/migration, EMT and tumor suppression by targeting several genes, including Smad7, TRIM14, Cyclin E1, MAP4K3, and IKKα (43, 44). In addition, we showed that PPARA was associated with the downregulation of mir-148a-3p whose overexpression has been reported to enhance M1 macrophage polarization leading to pro-inflammatory responses and enhanced ability to kill bacteria by targeting PTEN/AKT (45). Although expression levels of target genes or molecules of miRNAs differentially expressed in the UGT of vaccinated and infected mice was not evaluated in this study, we previously reported the altered expression of proteins that regulate epithelial functional integrity, EMT and tumorigenesis in the UGT following *Chlamydia* infection (8). Several of these proteins were identified to be targets of miRNAs that control epithelial integrity through EMT. Taken together, the results indicate that the up- or down-regulation of these miRs plays a significant role in the induction of innate and adaptive immune responses leading to protective immunity or immunopathology by regulating genes directly or indirectly associated with immunity and immunopathology.

In conclusion, we have demonstrated that vaccine immunization and *Chlamydia* infection induced the expression of distinct miRNA profiles in the mouse UGT and targeted genes that regulate several biological processes and functions associated with immune response and inflammation. The results strongly suggest that these unique miRNA profiles play a significant role in the disparate immunity outcomes associated with *Chlamydia* infection and vaccination through regulation of their target genes in combination with diverse biological processes and pathways. Further studies will investigate the molecular mechanisms linking these miRs with their gene targets and how they modulate immunity and immunopathology following *Chlamydia* infection and vaccination.

## Data Availability Statement

The original contributions presented in the study are included in the article/Supplementary Material, further inquiries can be directed to the corresponding author/s.

## Ethics Statement

The animal study was reviewed and approved by The Institutional Animal Care and Use Committee (IACUC) of Morehouse School of Medicine (MSM) (Assurance number A3381-01).

## Author Contributions

FE and YO conceived and designed the study. SH and SR performed most of the experiments. KD, FM, and SL contributed to the experimental work. IB, FE, and YO made substantial contributions to analysis and interpretation of data. OA generated Figure 1B. JI and YO contributed to data interpretation and critically revised the manuscript. FE wrote the manuscript. All authors have read and agreed to the publication of the manuscript.

## Conflict of Interest

The authors declare that the research was conducted in the absence of any commercial or financial relationships that could be construed as a potential conflict of interest.
