# Supplementary material for: Differential miRNA Profiles Correlate With Disparate Immunity Outcomes Associated With Vaccine Immunization and Chlamydial Infection
Source: Front Immunol. 2021 Feb 22;12:625318. doi: 10.3389/fimmu.2021.625318 (PMC7937703; doi:10.3389/fimmu.2021.625318)
Supplement: Supplementary file 1 [file Presentation_1.zip › Supplementary Figure Captions.PDF]

Differential miRNA profiles correlate with disparate immunity outcomes associated with vaccine immunization and chlamydial infection

Simone Howard[1]#, Shakyra Richardson[1]#, Ifeyinwa Benyeogor[2], Yusuf Omosun[1], Kamran Dye[3], Fnu Medhavi[1], Stephanie Lundy[1], Olayinka Adebayo[1], Joseph U. Igietseme[4] and Francis O. Eko[1*]

*** Correspondence:** Corresponding Author: feko@msm.edu

Supplementary Figures

Supplementary Figure 1. Positive Control Performance: Linearity of Counts vs. RNA Concentration. This graph demonstrates the linearity of the nCounter platform. The Pearson Correlation (R2) of target concentration vs. counts is plotted for the 6 Positive control probes across 12 assays. Correlations are in expected range (R2> 0.95).

Supplementary Figure 2. Differentially expressed miRNAs common between the different immunization groups by RT-PCR. Levels of select upregulated (miR-146a-5p, miR-126-3p) and downregulated (miR-15a-5p, miR-200c-3p) miRNAs differentially expressed in the upper genital tracts of mice immunized with DC vaccine or VCG vaccine or infected with live Chlamydia. The relative fold changes in miRNA expression were calculated by the delta-delta-cycle threshold (ΔΔCt) method comparing miRNA expression levels in the genital tracts after vaccines immunization and live Chlamydia infection. U6 miRNA was used as an endogenous control for expression normalization. ΔΔCt were calculated as the difference between immunized and non-immunized ΔCt. The results are expressed as fold change (the mean ± SD of three independent runs), corresponding to 2-ΔΔCt.

Supplementary Figure 3. Signaling pathways showing predicted gene targets of unique miRNAs differentially regulated in the upper genital tracts of mice after DC vaccination. (a) Inflammatory mediator regulation of TRP Channels signaling pathway, (b) P13K-AKT signaling pathway, (c) ECM-Receptor Interaction signaling pathway, (d) FOXO signaling pathway, (e) mTOR signaling pathway, (f) T cell Receptor signaling pathway, (g) HIPPO signaling pathway. Genes highlighted in yellow are predicted targets, while the upstream regulators that promote or antagonize each signaling pathway are indicated by orange or green, respectively. Arrowed or blunted ends indicate activation or inhibition, respectively. Continuous lines indicate known interactions, whereas dashed lines indicate unknown mechanisms.
