# Supplementary figures and images for: Differential miRNA Profiles Correlate With Disparate Immunity Outcomes Associated With Vaccine Immunization and Chlamydial Infection
Source: Front Immunol. 2021 Feb 22;12:625318. doi: 10.3389/fimmu.2021.625318 (PMC7937703; doi:10.3389/fimmu.2021.625318)

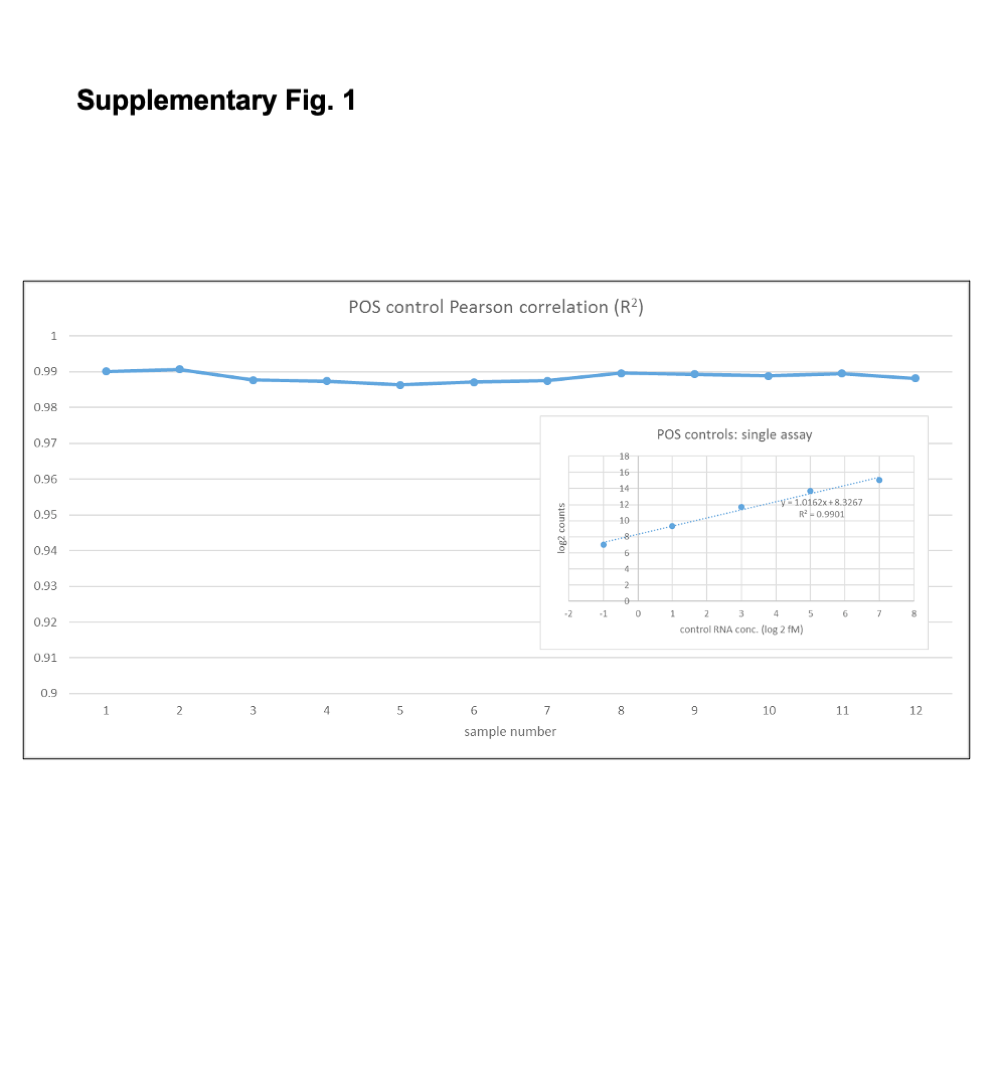

Supplement: Supplementary file 1 [file Presentation_1.zip › Figure S1.TIFF]

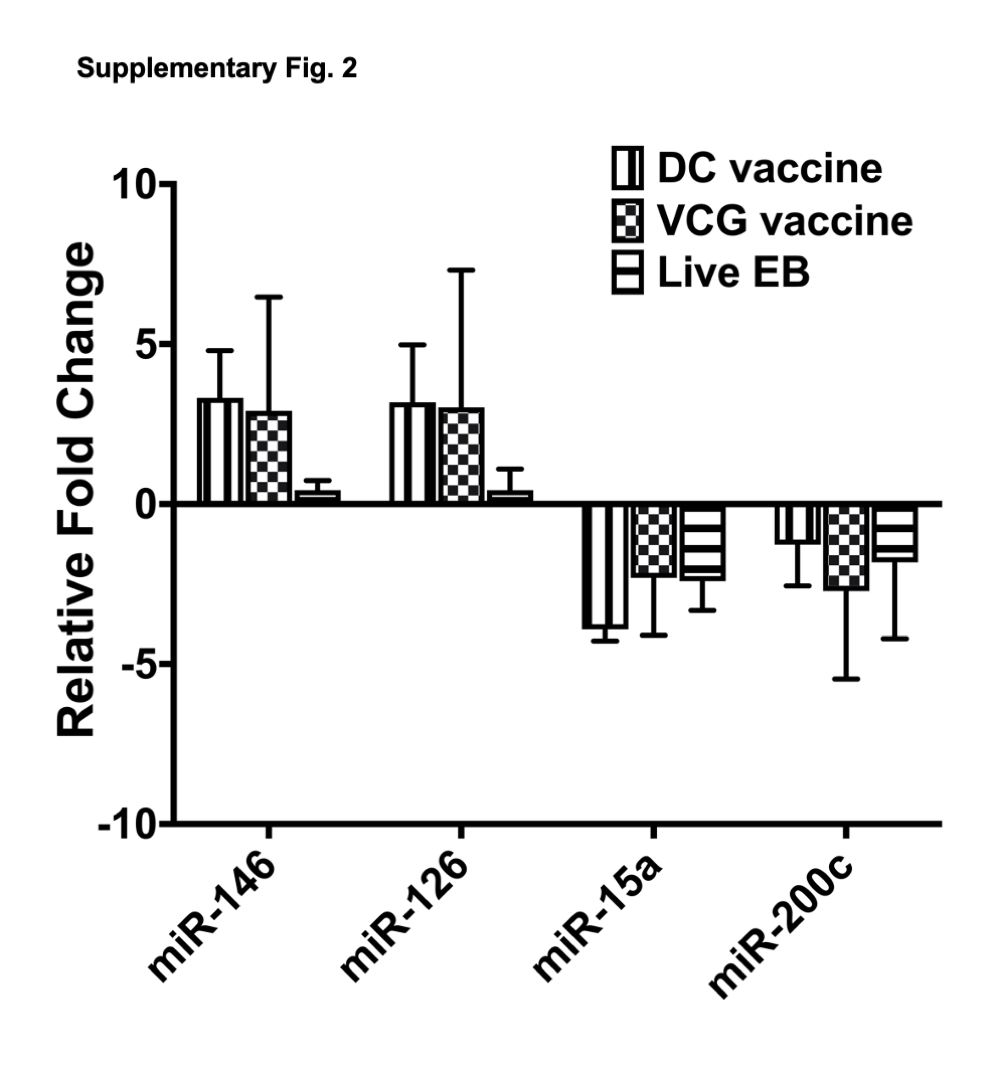

Supplement: Supplementary file 1 [file Presentation_1.zip › Figure S2.TIFF]

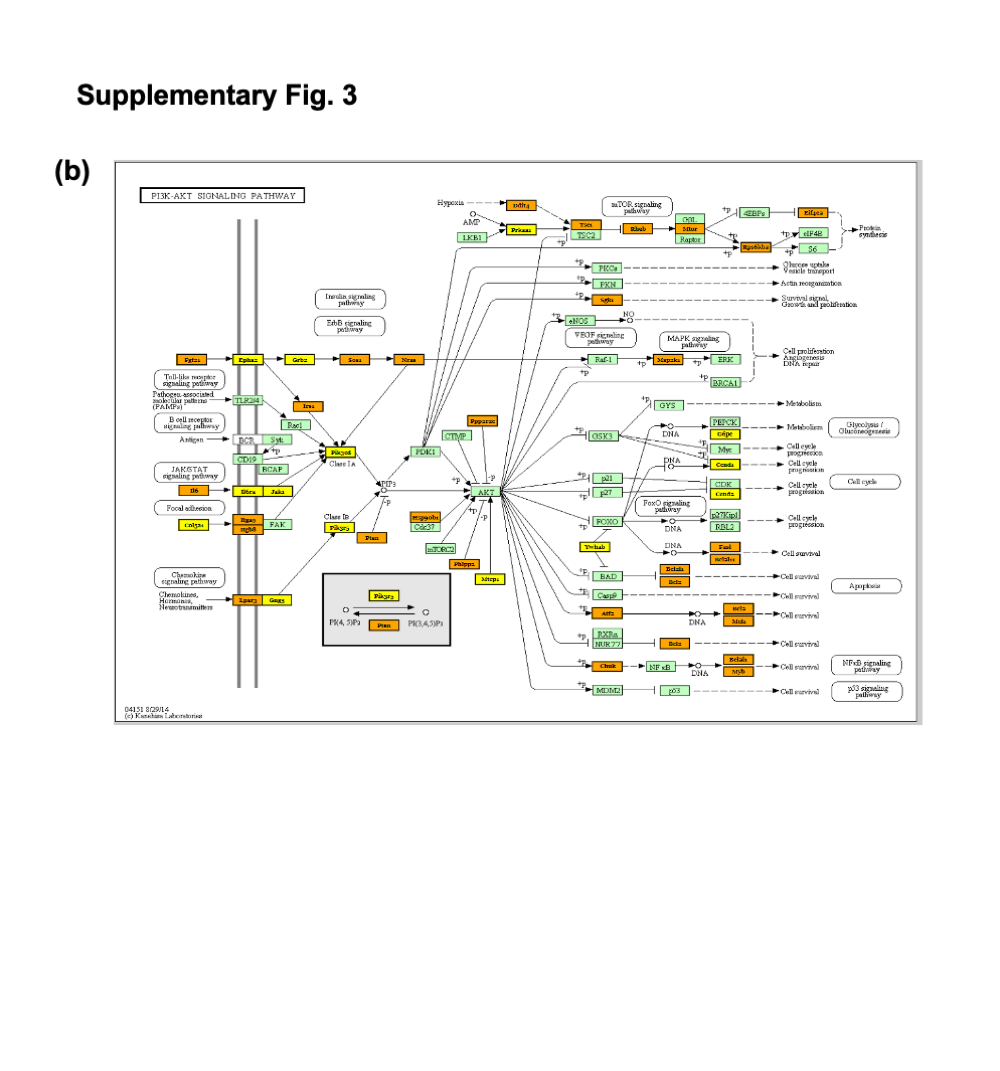

Supplement: Supplementary file 1 [file Presentation_1.zip › Figure S3 b.TIFF]

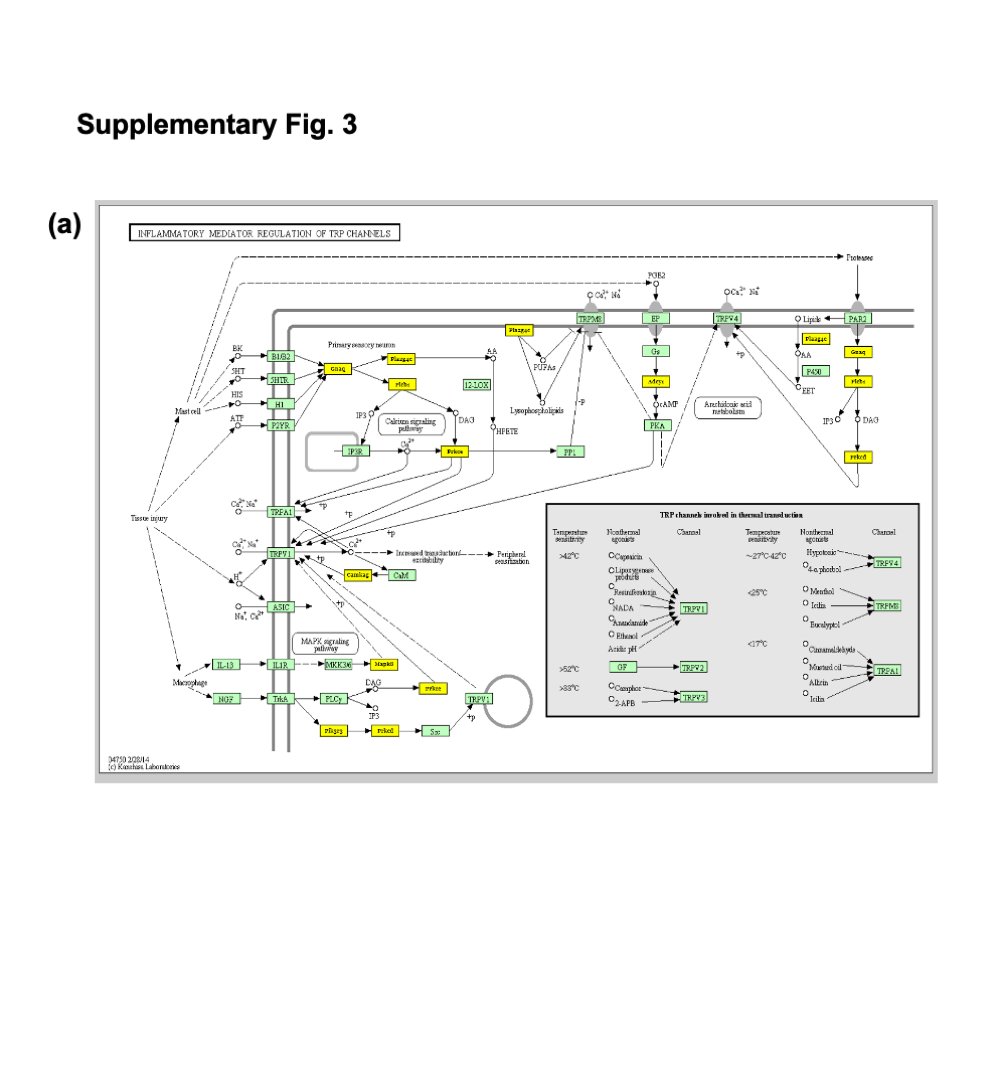

Supplement: Supplementary file 1 [file Presentation_1.zip › Figure S3a.TIFF]

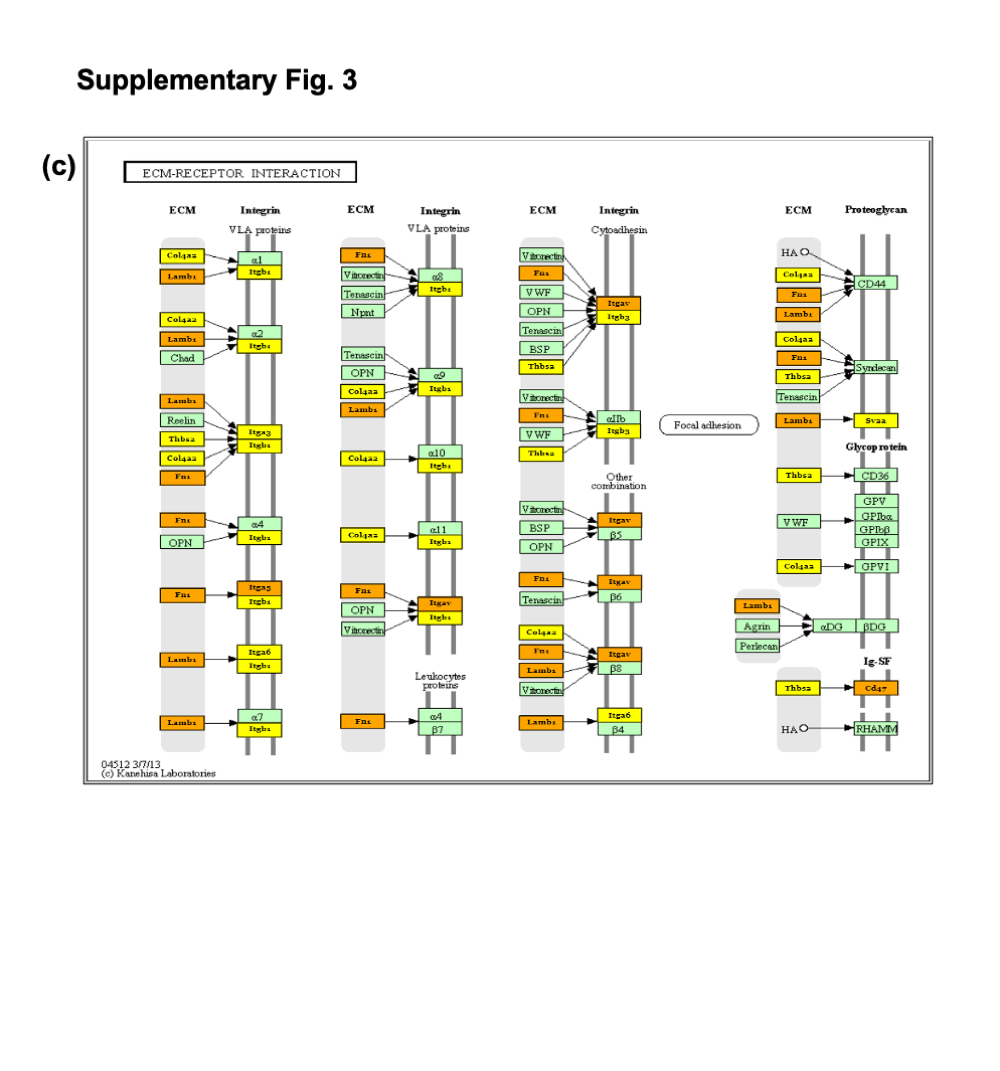

Supplement: Supplementary file 1 [file Presentation_1.zip › Figure S3c.TIFF]

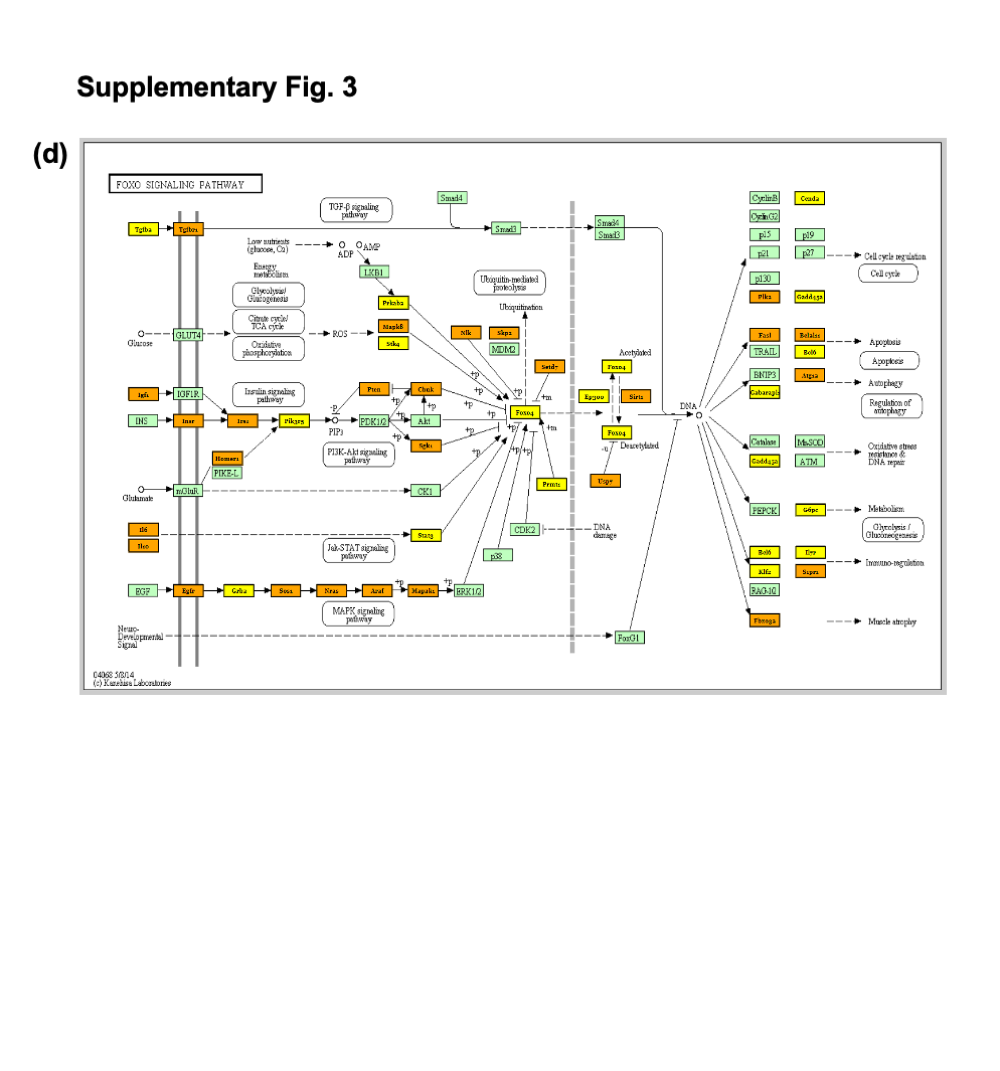

Supplement: Supplementary file 1 [file Presentation_1.zip › Figure S3d.TIFF]

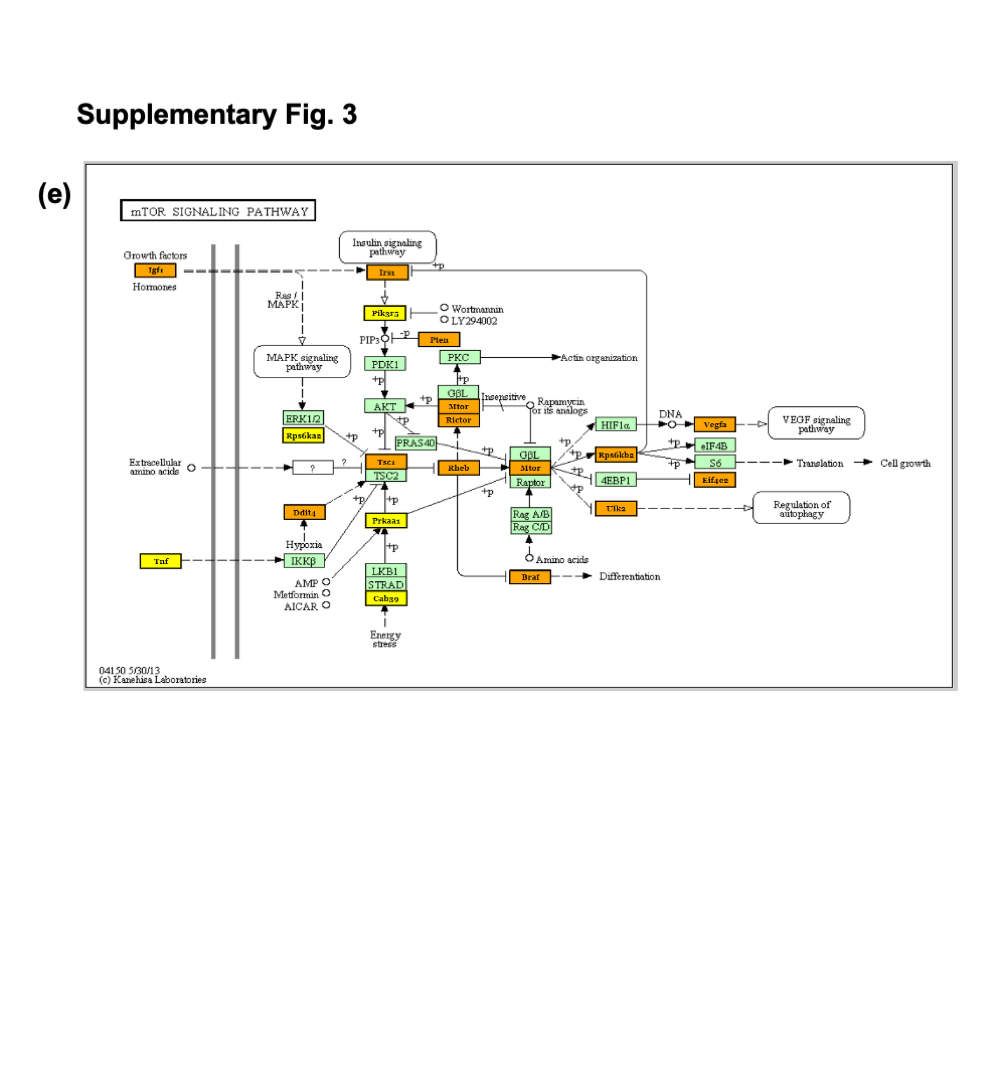

Supplement: Supplementary file 1 [file Presentation_1.zip › Figure S3e.tiff]

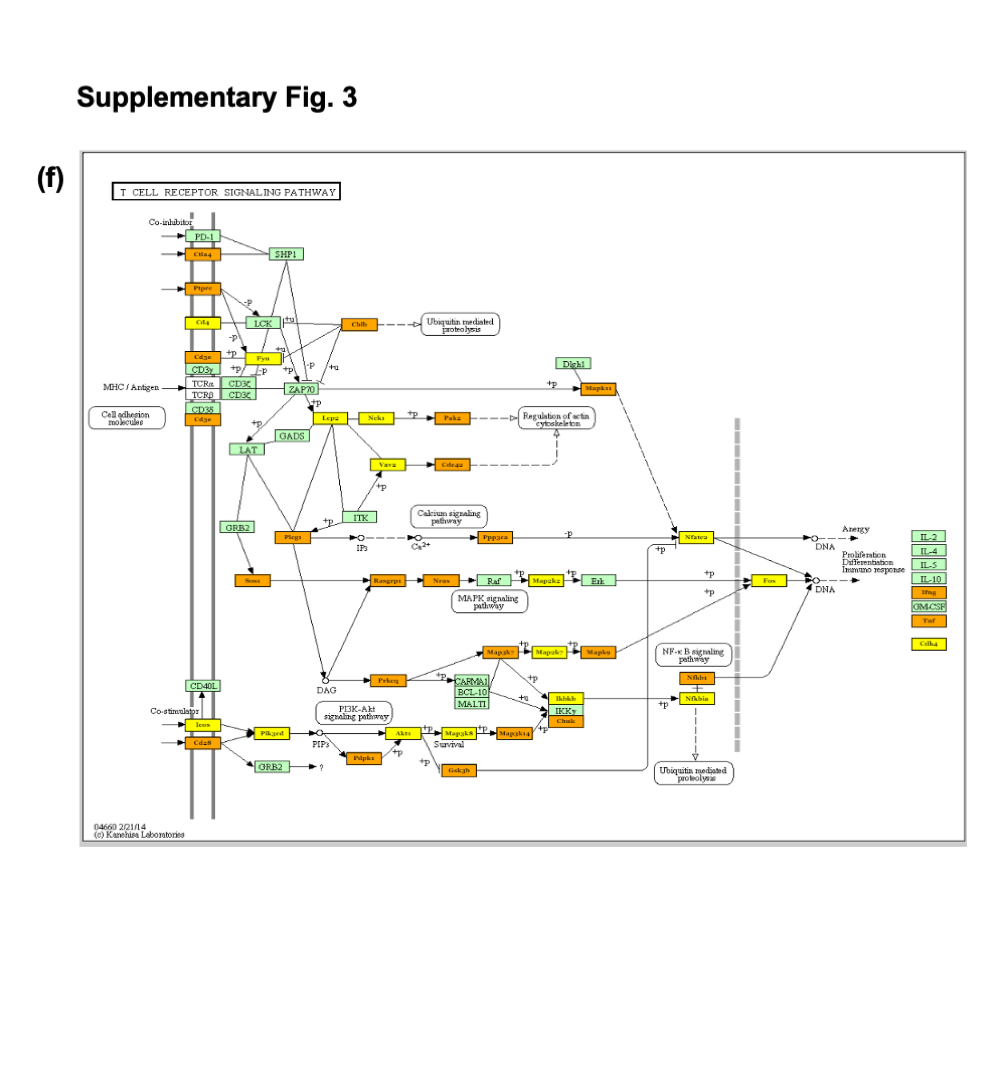

Supplement: Supplementary file 1 [file Presentation_1.zip › Figure S3f.TIFF]

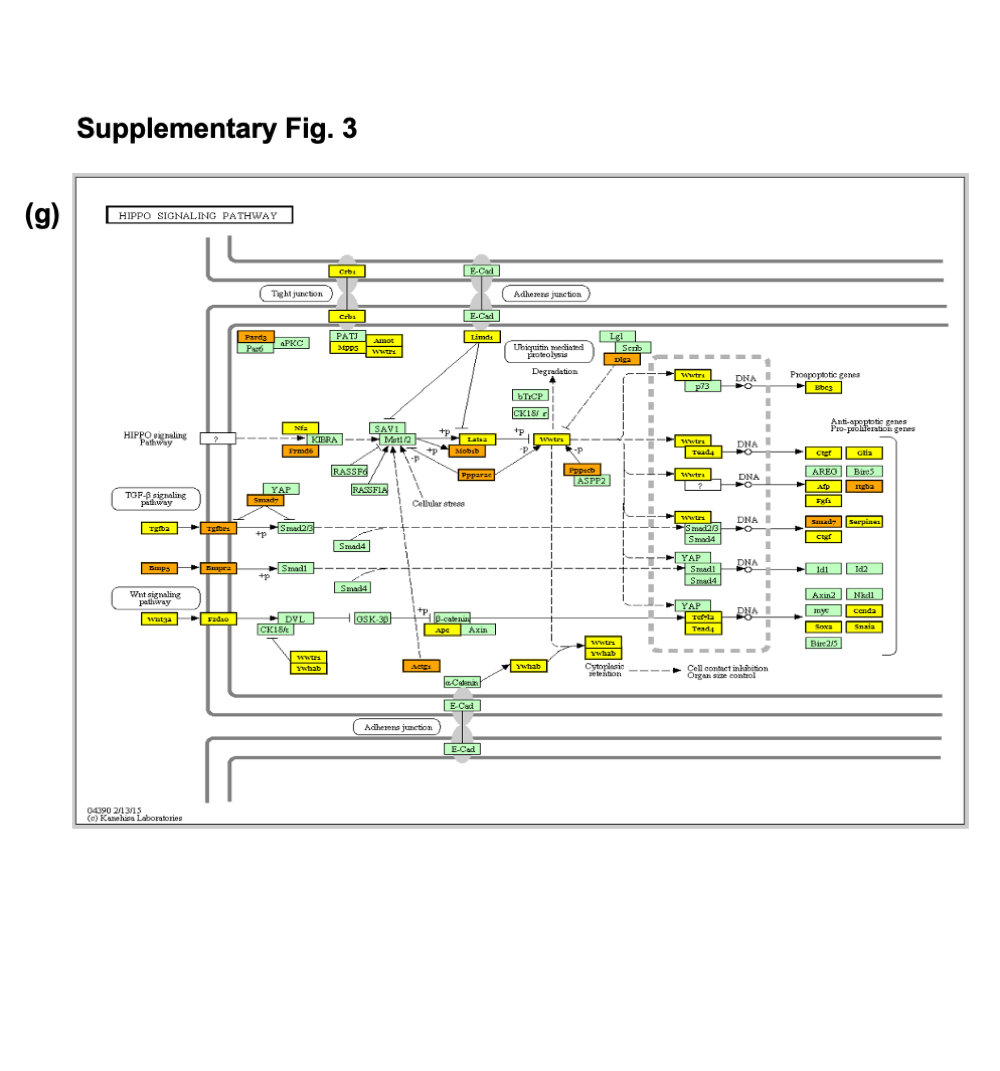

Supplement: Supplementary file 1 [file Presentation_1.zip › Figure S3g.TIFF]
